# Duck virus enteritis (duck plague) outbreak in an Australian black swan (*Cygnus atratus*) flock at safari park in Bangladesh: A case report

**DOI:** 10.5455/javar.2021.h545

**Published:** 2021-10-12

**Authors:** Md. Mohirul Islam, Jahidul Islam, Md. Sadequl Islam, Tanvir Ahamed, Mohammad Rafiqul Islam, Mst. Minara Khatun, Md. Ariful Islam

**Affiliations:** 1 Department of Microbiology and Hygiene, Faculty of Veterinary Science, Bangladesh Agricultural University, Mymensingh, Bangladesh; 2 Department of Anatomy and Histology, Faculty of Veterinary and Animal Science, Hajee Mohammad Danesh Science and Technology University, Dinajpur, Bangladesh; 3 Livestock Division, Bangladesh Agricultural Research Council, Dhaka, Bangladesh

**Keywords:** Australian black swan, Bangladesh, duck viral enteritis virus, phylogenetic analysis, safari park

## Abstract

**Objective:**

Duck virus enteritis is a severe viral disease that kills ducks and swans worldwide. The clinical manifestations, gross pathology, molecular detection, and characterization of the duck virus enteritis virus (DVEV) in Australian black swans at a safari park in Bangladesh were described in this case report.

**Materials and Methods:**

On a safari park in Bangladesh, an Australian black swan flock exhibited clinical signs of anorexia, greenish watery diarrhea, increased thirst, partial paralysis, and death. Postmortem examinations of deceased swans revealed extensive pathological abnormalities in the trachea, liver, and spleen. To isolate DVEV, a viral inoculum produced from the liver and spleen of dead swans was implanted into 9–13-day-old embryonated duck eggs via the chorioallantoic membrane (CAM) route. DVEV was confirmed using a polymerase chain reaction (PCR) assay. Phylogenetic analysis was used to determine the genetic relationship between the DVEV isolates from Australian black swans, and 16 DVEV isolates previously described in the GenBank.

**Results:**

Hemorrhage was noted in the annular ring of the trachea, as well as an enlarged and hemorrhagic liver and spleen. The PCR assay amplified a 446-bp fragment of the DVEV DNA polymerase gene in the liver, spleen, and CAM homogenates. The phylogenetic analysis found that the DVEV isolates from swans were comparable to those from Bangladesh, India, Vietnam, China, Germany, the USA, and Egypt.

**Conclusion:**

According to the findings of this study, the DVEV was the cause of illness and mortality in an Australian black swan flock.

## Introduction

Duck virus enteritis (DVE), often known as duck plague, is caused by a double-stranded DNA virus under the family Herpesviridae [1]. Extreme thirst, watery greenish diarrhea, dehydration, paresis, trembling, and abrupt death of ducks are all symptoms of the disease [2]. Infected ducks exhibit extensive bleeding in the body cavities, gut, spleen, liver, heart, and pericardium [3]. DVE is diagnosed based on a history of illness outbreaks, clinical symptoms, and gross lesions in the ducks’ affected organs. Confirmatory diagnosis, on the other hand, requires virus isolation and identification. For the detection of duck virus enteritis virus (DVEV), polymerase chain reaction (PCR)-based molecular techniques are sensitive and accurate [4].

DVE has been detected in several countries that produce ducks, including Canada, England, Hungary, Denmark, Austria, Belgium, China, Vietnam, India, and Thailand [2]. It is a disease that affects both wild and domestic waterfowls, such as ducks, geese, and swans [2,5]. Domestic ducks are primarily affected by the epidemic in a location adjacent to aquatic settings co-habiting with wild waterfowl [6,7]. Migratory waterfowl aid in the transmission of information within and between continents. Although the consumption of infected water is the primary transmission mechanism, the virus can also be transferred via contact [6].

Wild ducks, swans, graylag geese, tundra bean geese, and grey herons have a high incidence of DVEV and may act as a carrier in neighboring water bodies [7]. Even recovered birds can serve as reservoirs for DVEV for up to a year, allowing the virus to propagate among susceptible waterfowl and contaminated settings [8,9]. Scavenging and decay of DVEV-infected carcasses can contaminate soil and water, hence spreading the virus throughout the environment. Numerous findings indicate that DVEV is transmitted via contaminated bird eggs [2,9].

Worldwide commerce, including international trade in live animals and animal products, is almost certainly the primary source of new disease outbreaks among wildlife inhabitants. Imported live animals are mostly used for commercial breeding, zoo and animal park operations, and laboratory research. The ongoing disintegration of barriers between livestock and wild animals facilitates the transmission of disease into livestock. Frequent interaction between diverse populations may result in the transmission of infections. Duck plague began in domestic birds in North America and quickly spread to wild ducks [10]. The DVE was initially identified in 1980 in Bangladesh [11]. The disease is common in Bangladesh, killing between 60% and 75% of the duck population [12]. DVE outbreaks have been documented in captive ducks and swans at zoos and recreational areas [13–15].

In 2018, a possible DVE epidemic was discovered in a flock of Australian black swans in a Bangladesh safari park. The current report discussed the clinical symptoms, gross pathology, isolation, and characterization of DVEV from DVE-infected Australian black swans. 

## Material and Methods

In March 2018, a suspected DVE outbreak was reported in an Australian black swan flock at a safari park in Bangladesh, located at latitude 24.1716663°N and longitude 90.3926611°E. The affected swan flock manifested clinical signs of anorexia, diarrhea, partial paralysis, and death, similar to infection caused by DVEV. The swan flock size was 20, which were reared in a lake of the safari park. The migratory waterfowls and other wild birds were frequently seen along with the swan flock. The swan flock was kept under a semi-intensive husbandry system with feed supplementation. 

Dead swans were collected and packed in an icebox and transported to the laboratory for postmortem examination and virological analysis. Clinical signs of the affected swans were recorded. Spleen and liver samples were aseptically collected from dead Australian black swans. Gross pathological lesions of the visceral organ such as liver, spleen, and trachea were recorded. 

To isolate DVEV from dead swans, a 10% tissue homogenate was prepared from the liver and spleen in PBS and centrifuged at 10,000 rpm for 10 min [16]. The sterile viral inoculum was inoculated into 9–13-day-old embryonated duck eggs through the chorioallantoic membrane (CAM) route [16] and incubated for 5–8 days at 37°C in an egg incubator. Embryos that died after 24 h were chilled. The CAM from the dead embryo was collected. 

According to the manufacturer’s instruction, the DNA was extracted from CAM homogenate by a commercial DNA extraction kit (Gene Proof, Brno, Czech Republic). A previously described PCR assay targeting 446-bp fragments of the DNA polymerase gene of DVEV was carried out for molecular detection of DVEV [17].

A phylogenetic study was carried out for determining the genetic relationship of DVEV of Australian black swans with global DVEV available in the GenBank. The partially amplified DNA polymerase gene product (446-bp) was sequenced from a commercial company (Apical Scientific Selangor, Malaysia). The DNA sequences were aligned with 16 published sequences of DVEV reported in the GenBank, and a phylogenetic tree was constructed by the maximum likelihood method using MEGA X [18].

## Results

The clinical signs observed in Australian black swans included depression, ruffled feathers, ocular and nasal discharge, anorexia, labored breathing, greenish watery diarrhea, extreme thirst, and ataxia, followed by death. The morbidity and mortality rates were 50% (10 of 20) and 30% (6 of 20), respectively.

**Figure 1.**
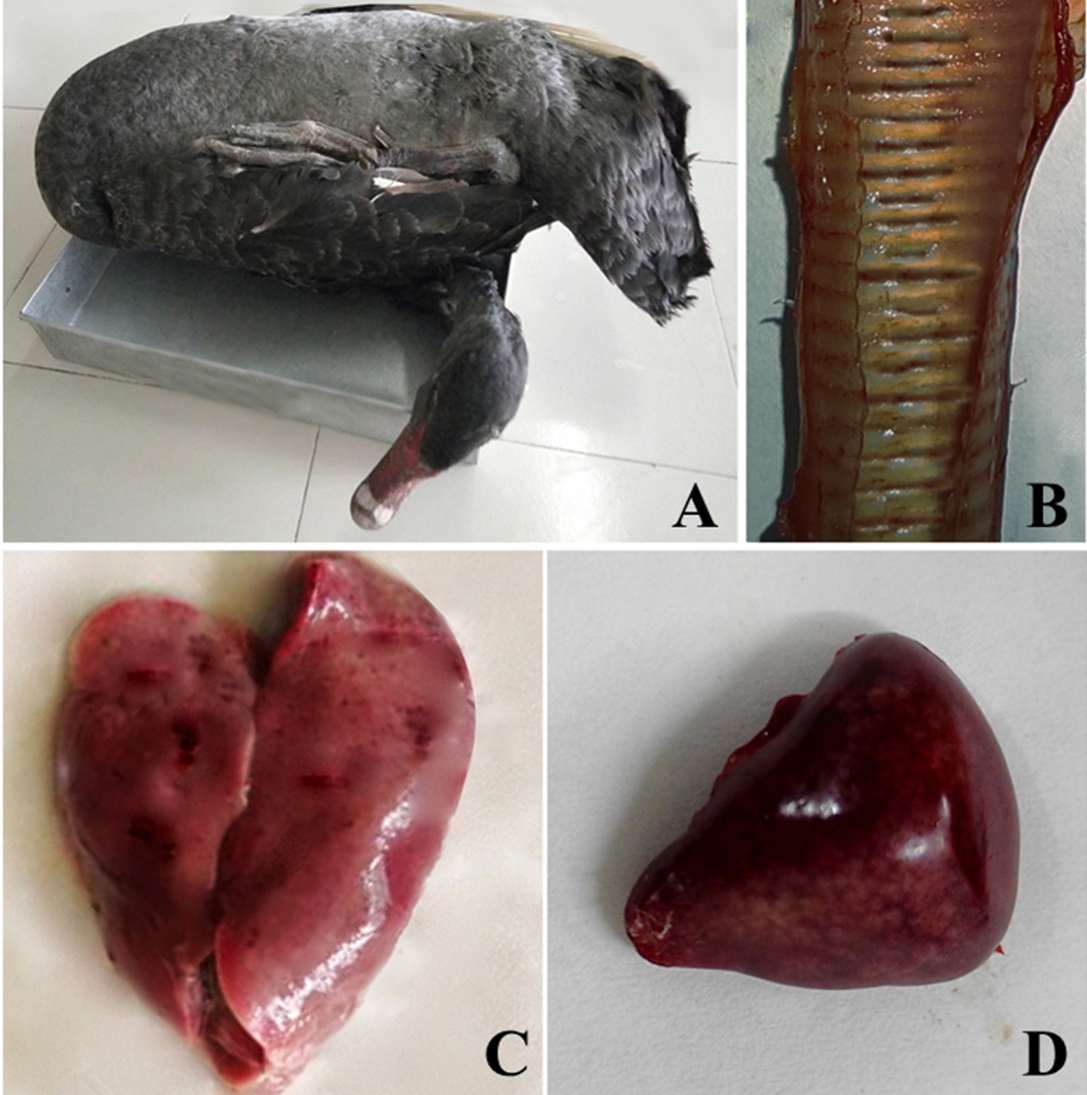
Gross pathological lesions of a dead Australian black swan suspected to be infected with DVE. A dead Australian black swan (A), hemorrhage on the annular tracheal bands (B), pale and enlarged liver with pin point hemorrhages on its surface (C), and dark enlarged spleen with numerous petechial hemorrhages on its surface (D).

During postmortem examination, the gross pathological lesions observed in dead Australian black swans included enlarged hemorrhagic liver and spleen and hemorrhage of the annular tracheal bands (Fig. 1).

**Figure 2.**
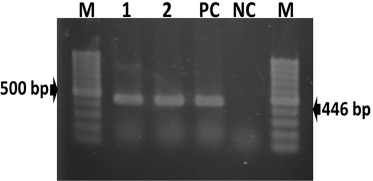
Amplification of a partial fragment of DNA polymerase gene (446-bp) of duck viral enteritis virus by PCR. Lane M: 100 bp size DNA ladders (Thermo Scientific, USA); Lanes 1–2: DNA extracted from samples of Australian black swans; Lane PC: positive control; and Lane NC: negative control.

The DNA extracted from CAM homogenate were successfully amplified in PCR assay with the production of a 446-bp fragment of DNA polymerase gene (Fig. 2).

**Figure 3.**
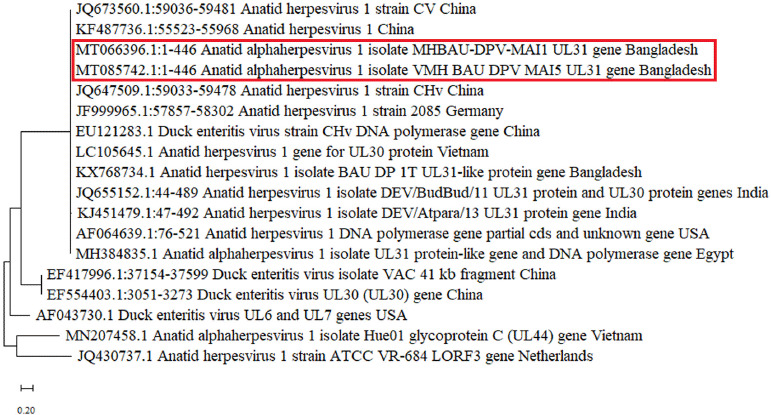
Phylogenetic analysis based on partial sequences (446-bp) of DNA polymerase gene of DVEV isolates from 16 reference strains obtained from GenBank. There are two sequences reported in the tree from this study (MT066396 and MT085742) that are found similar to the DVEV previously reported in Bangladesh, India, Vietnam, China, Germany, the USA, and Egypt. The tree is constructed by the maximum likelihood method using MEGA X software.

The phylogenetic tree derived from the sequence data is shown in Figure 3. The phylogenetic tree showed that sequenced strains of the two DVEVs of this study (GenBank accession no. MT066396 and MT085742) were similar to Anatid herpesvirus 1 isolates previously reported from Bangladesh, India, Vietnam, China, Germany, USA, and Egypt.

## Discussion

DVE is a viral illness that is lethal to ducks and swans in Southeast Asia [19]. DVE causes significant economic losses in commercial, domestic duck farms due to carcass condemnation and decreased egg production [20]. The current study evaluated a possible DVE outbreak in Australian black swans in a safari park.

DVE is highly lethal to both domestic and wild ducks [2]. The DVE produces death and morbidity rates of up to 100% in ducks and swans [9], depending on the severity of the infecting virus and the duck’s immunological condition [20]. The recent investigation discovered a 30% death rate among Australian black swans. Keymer and Gough [21] reported a 22.5% mortality rate in DVEV-infected captive mute swans, with substantial clinical symptoms including polydipsia, photophobia, wing drooping, inappetence, watery nasal discharge, and yellowish diarrhea [21]. Clinical symptoms almost identical to those seen in the affected Australian black swans were observed in this investigation. Viral replication results in increased vascular permeability, which results in lesions and hemorrhages in the spleen, thymus, liver, and bursa of Fabricius [22,23]. Postmortem lesions almost identical to those observed in the reported Australian black swans were also observed. Clinical symptoms and gross pathological lesions observed in this study’s DVE-infected flock and other flocks described by numerous investigators vary according to the afflicted birds’ species, immunological status, age, and sex, as well as the virulence of the field virus strain [24].

Migratory waterfowls are the key risk factors for disease dissemination [9,25,26]. Ducks that were sick or recovered transmit viruses through their droppings, polluting nearby bodies of water [8]. The viruses can survive and stay for an extended period of time in dirty, stagnant, and slow-moving water bodies [24]. A susceptible duck may contract DVEV by oral, nasal, or cloacal routes, as well as through skin wounds [2]. The suspected swan flock may have contracted the virus by drinking or eating in the safari park lake’s tainted water. Wild birds have also been observed to carry the virus that causes this disease to spread to susceptible flocks. DVE outbreaks have been recorded often in duck flocks that cohabitate with free-range waterfowl [9]. During the spring season, from March to June, outbreaks of DVE are regularly observed in susceptible duck and swan flocks [2]. During the spring season, convalescent ducks were reported to release the virus spontaneously [2]. DVE outbreaks occurred in March in the reported swan flock. Exotic migrating ducks freely interacted and scavenged with the lake’s swan flock. Numerous tourists approached the premises of the safari park’s swan flock and were frequently provided food. Wild birds were spotted in the woods surrounding the safari park’s lake. DVEV has been detected in wild birds [27].

A phylogenetic analysis was conducted to assess the genetic link between the DVEV isolates and those in the GenBank. The phylogenetic study revealed that the sequenced strains of DVEV were highly similar to DVEV isolates from Bangladesh, India, Vietnam, China, Germany, the USA, and Egypt. Ahamed et al. [28] reported a genetic similarity between Bangladeshi DVEV isolates and DVEVs from China.

## Conclusion

This study established that DVEV was the cause of death in an Australian black swan flock at a safari park. To combat this lethal viral infection, regular surveillance of DVEV in swan flocks, effective immunization, and biosecurity protocols must be implemented.

## List of abbreviations

CAM, chorioallantoic membrane; DVE, duck virus enteritis; DVEV, duck virus enteritis virus; PCR, polymerase chain reactions; PBS, Phosphate-buffer solution.
